# The battle between bacterial infection and autophagy in aquatic animals

**DOI:** 10.3389/fimmu.2025.1614182

**Published:** 2025-06-23

**Authors:** Qi Wang, He Xu, Jiaxue Jin, Yankai Yang, Lothar Jänsch, Senlin Li

**Affiliations:** ^1^ School of Life Sciences, Zhengzhou University, Zhengzhou, China; ^2^ Cellular Proteomics, Helmholtz Centre for Infection Research, Braunschweig, Germany

**Keywords:** autophagy, xenophagy, immunology, teleost, bacteria

## Abstract

Autophagy is a conserved cellular degradative pathway that has been demonstrated to play a crucial role in the innate immune response to combat infection with a range of pathogenic bacteria via xenophagy. Although this process has been well-described in terrestrial animals, the extent to which autophagy contributes to aquatic animal-bacteria interactions remains poorly understood. Autophagy can directly eliminate intracellular pathogens by acting as a conduit for their lysosomes delivery. Consequently, bacteria have evolved a variety of tactics to evade autophagy. This is accomplished by interfering with autophagy signaling or the autophagy machinery itself. In certain instances, bacteria even utilize autophagy as a means of promoting their growth. This review discusses canonical and non-canonical autophagy pathways and current knowledge of autophagy in aquatic animals. This review illuminates the intricate relationship between autophagy components and intracellular bacteria. It explores how the autophagic machinery senses these bacteria directly or indirectly, the interaction between autophagy and effectors/toxins secreted by bacteria, and how some of these bacterial pathogens evade autophagy.

## 1 Introduction

**Figure 1:**
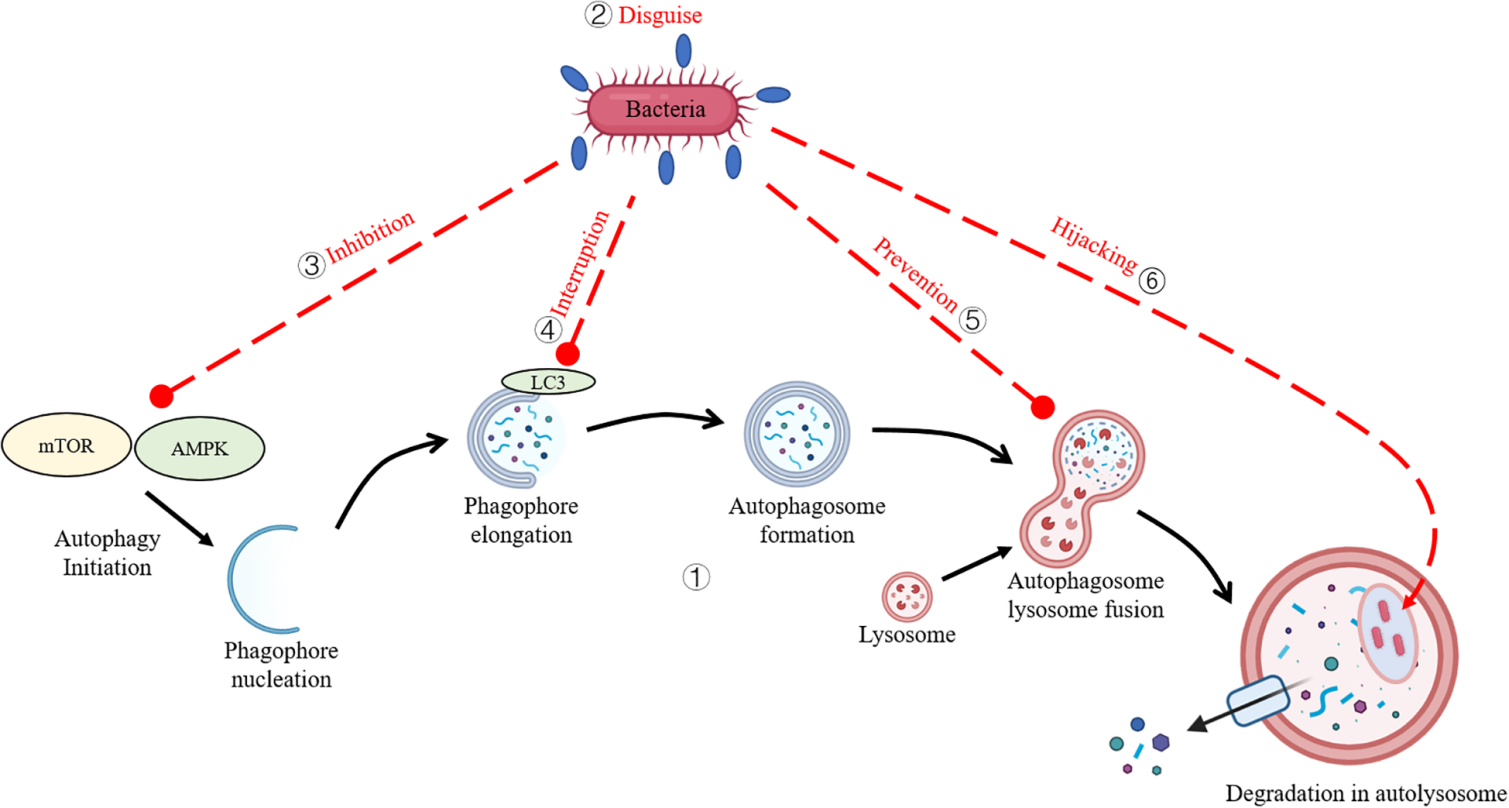
Bacterial Strategies to Evade Autophagy. (1) The core autophagy pathway in teleost species. (2) Bacteria disguise their surface antigens by coating them with the host’s own proteins. (3) Bacteria subvert host autophagy by inhibiting upstream signaling cascades. (4) Bacteria interfere with autophagic proteins to evade capture. (5) Bacteria inhibit autophagosome-lysosome fusion. (6) Bacteria exploit autophagy machinery to create intracellular replicative niches.

Autophagy constitutes a highly conserved “clean-up” process in eukaryotic organisms, essential for maintaining intracellular homeostasis through the break down and recycle unnecessary or damaged materials like long-lived proteins, aggregated cellular components, and superfluous or dysfunctional organelles, including mitochondria and peroxisomes. This process is categorized into three primary subtypes: macroautophagy (where the cell wraps debris in a membrane-bound sac), microautophagy (where the lysosome directly engulfs material), and chaperone-mediated autophagy (which uses helper proteins to shuttle specific cargo to the lysosome). Furthermore, emerging evidence highlights autophagy as an integral component of innate immunity, particularly through xenophagy, a specialized form of selective autophagy, which enabling the recognition, encapsulation, and lysosomal degradation of harmful bacteria, viruses, or other invaders inside the cell, thereby limiting their proliferation and mitigating infection. This interplay underscores autophagy’s dual role in cellular maintenance and protect organisms by preventing pathogens from hijacking cellular resources (1, 2). Growing evidence has elucidated that bacterial pathogens have evolved sophisticated counterstrategies to subvert autophagy (3) (as shown in **Figure 1**).

For example, some bacteria block the signals that trigger autophagy by inhibiting upstream signaling cascades (e.g., mTOR or AMPK pathways) (4, 5), disguise their surface antigens using the host’s own proteins to evade being flagged for destruction, or mess with the proteins (e.g., ATG5, LC3) responsible for autophagy to dodge capture (6–8). Additionally, certain species obstruct autophagosome-lysosome fusion, thereby preventing pathogen degradation (9). Shockingly, a few bacteria hijack parts of the autophagy system to establish replicative niches, enhancing intracellular survival (10). While extensive research has illuminated the interplay between autophagy and bacterial pathogens in mammalian systems, this topic hasn’t gotten nearly as much attention in aquatic organisms. Notably, review addressing autophagy-pathogen interactions in aquatic species are scarce, reflecting a critical gap in the field. This review dives into the molecular interplay between autophagy and intracellular pathogens, elucidating the mechanisms by which xenophagic pathways target invasive bacteria and the sneaky tactics pathogens employ to evade detection, avoid autophagic capture, or inhibit lysosomal degradation. This review compiles publications from 1997 to 2025, focusing on bacterial interactions with host autophagy and their evasion strategies in teleost. Additionally, some bacteria known to infect mammals are included to provide broader insights into bacterial evasion mechanisms. By piecing together emerging evidence, this work sheds light on the evolutionary adaptations of pathogens, offering insights into potential therapeutic avenues for aquatic disease management.

## 2 Autophagy

### 2.1 Autophagy classification

Canonical autophagy, mediated by a conserved suite of autophagy-related proteins, involves double-membrane autophagosome formation. A hallmark of this process is the recruitment of microtubule-associated protein 1 light chain 3 (LC3), a canonical autophagosome marker. Phagosomes—vesicles mediating extracellular particle engulfment—recruit LC3 via LC3-associated phagocytosis (LAP) (11), a distinct pathway sharing lysosomal degradation aims but diverging in structure and origin. While autophagosomes sequester cytoplasmic cargo within double membranes, LAP involves LC3 conjugation to single-membrane phagosomes (12). Notably, Rubicon, essential for LAP, is dispensable in canonical autophagy targeting intracellular pathogens, highlighting the functional plasticity of autophagy machinery in homeostasis and immunity (13). Autophagy operates via non-selective and selective modalities. Non-selective autophagy indiscriminately engulfs cytoplasmic components en masse for energy recycling. In contrast, selective autophagy employs molecular tagging (e.g., ubiquitination) and adaptor-mediated cargo recognition to precision-target substrates—including mitochondria (mitophagy), ribosomes (ribophagy), peroxisomes (pexophagy), and pathogens (xenophagy)—for lysosomal degradation (14) (as shown in **Table 1**). This review focuses on xenophagy, delineating its antimicrobial mechanisms and bacterial evasion tactics.

**Table 1:** Classification and key differences between autophagy pathways.

Items	Two-tiered classification	Three-tiered classification	Membrane structure	Trigger	Function	Regulatory proteins	target
LC3-associated phagocytosis (LAP)			Single-membrane	Receptor signaling	Immune regulation	Rubicon, NOX2	Extracellular particles
Canonical autophagy	Non-selective autophagy		Double-membrane	Nutrient insufficiency	Metabolite recycling	Various ATG proteins	Random cytoplasmic content
	Selective autophagy	Mitophagy	Double-membrane	Mitochondrial damage, PINK1/Parkin pathway, nutrient starvation	Mitochondrial quality	PINK1/Parkin pathway	Mitochondria
		Ribophagy		Nutrient starvation, unfolded protein response, Ribosome damage	Protein synthesis regulation	Autophagy receptors	Ribosomes
		Pexophagy		Peroxisome dysfunction, nutrient starvation, reactive oxygen species	Lipid metabolism and detoxification	Autophagy receptors	Peroxisomes
		Xenophagy		Intracellular pathogen detection, pattern recognition receptors, inflammatory signals	Immune defense	Autophagy receptors	Intracellular pathogens

### 2.2 Xenophagy targeting mechanisms

Host cells deploy cytosolic surveillance systems to detect invasive pathogens via pathogen- and damage-associated molecular patterns (PAMPs/DAMPs), which activate pattern recognition receptors (PRRs) such as NOD-like receptors. These receptors trigger innate immune responses, enabling detection of both microbial presence and infection-induced cellular stress (15, 16). And then, xenophagy, a selective autophagy pathway, targets intracellular pathogens for lysosomal degradation, serving as a key antimicrobial mechanism (17, 18). Pathogens are categorized by their intracellular niches: those confined within vesicles or replicating freely in the cytosol (19). Typically, Rapid bacterial clearance occurs unless pathogens evolve evasion strategies (20). Among autophagy-mediated targeting mechanisms, those involving protein ubiquitination remain the most extensively characterized. Ubiquitin chains play a pivotal role in autophagy, functioning as molecular signals that direct bacterial clearance. During infection, ubiquitin orchestrates the autophagic sequestration of invasive bacteria by mediating interactions between pathogen-containing endosomes and the autophagic machinery upstream of LC3 recruitment. Ubiquitination is central to autophagy, with E3 ligases (e.g., PRKN, SMURF1) depositing ubiquitin chains on pathogens to recruit autophagy adaptors (SQSTM1/p62, NDP52, optineurin), which bridge cargo to nascent autophagosomes. These adaptors bridge ubiquitinated cargo to LC3-decorated autophagosomes via ubiquitin-binding and LC3-interacting domains (21, 22). TBK1-mediated phosphorylation of autophagy receptors enhances their recruitment to bacteria and autophagosome formation during autophagy (23). Emerging evidence underscores ubiquitin’s direct interaction with Atg16L1, bypassing LC3 to streamline pathogen recognition and degradation, reinforcing ubiquitin’s pivotal role in autophagy (24–26).

## 3 Evasion of xenophagy by intracellular bacteria

Xenophagy primarily functions to eliminate intracellular pathogens by detecting and tag them within autophagosomes, which subsequently fuse with lysosomes to mediate pathogen degradation. However, numerous bacterial pathogens have developed counterstrategies to subvert xenophagic clearance, enabling their survival and replication within host cells.

### 3.1 Avoidance of autophagy recognition

Xenophagy targets cytosolic bacteria via host ubiquitin machinery, which deposits polyubiquitin coats on pathogens, enabling recognition by autophagy receptors (e.g., p62, NDP52) (27). Autophagosome formation is initiated by BECLIN 1, with LC3 lipidation via the ATG5–12–16L1 complex enabling bacterial capture (28, 29). Subsequent lysosomal fusion mediates degradation. During infection, intracellular bacteria prompt the formation of ubiquitinated aggregates (e.g., vacuolar remnants, bacterial debris, aggresome-like structures), which recognized by ubiquitin-binding proteins (30). But, a hallmark of many intracellular pathogens, including *Salmonella enterica*, who replicates within a membrane-bound compartment, the *Salmonella*-containing vacuole (SCV), that counteracts this by deploying the SPI-2 type III secretion system (T3SS) effector SseL, a deubiquitinase, reducing autophagic marker recruitment and enhancing bacterial replication within SCVs (31, 32).

The Gram-positive bacterium *Listeria monocytogenes* (*L. monocytogenes*) is a facultative intracellular pathogen capable of invading and replicating within mammalian or teleost cells (33). Upon entering the cytosol, it employs the surface protein ActA to evade autophagic recognition. ActA’s amino-terminal domain binds and activates the host Arp2/3 complex to drive actin nucleation, while its central proline-rich region recruits Ena/VASP proteins to enhance bacterial motility (34). By recruits host cytoskeletal components (e.g., Arp2/3, VASP, Actin), ActA orchestrates actin polymerization, disguising the bacterium as a host organelle and preventing detection by autophagy markers such as polyubiquitin, p62, and LC3 (35). Additionally, the protein InlK further shields *L. monocytogenes* by interacting with the host major vault protein, blocking ubiquitin tagging by E3 ligases and autophagy receptor recruitment (36).

*Shigella flexneri* (*S. flexneri*), a Gram-negative bacterium causing shigellosis, evades xenophagy via its T3SS effector IcsB and surface protein IcsA. Autophagy protein ATG5 binds IcsA, which normally targets pathogens for degradation, and promotes actin polymerization. This indirectly supports the formation of septin cages—cytoskeletal structures that, alongside autophagy proteins, trap *Shigella* to limit spread (37). Ubiquitin binding adaptor proteins p62 and NDP52 then direct the bacteria to septin- and actin-dependent autophagy pathways (38). However, IcsB competitively blocks ATG5 binding to IcsA by occupying the same IcsA domain (amino acids 320–433) in a dose-dependent manner, preventing autophagosome recognition (39).

*Rickettsia* species are Gram-negative, obligate intracellular bacteria that infect vascular endothelial cells, dendritic cells, and macrophages. *R. parkeri* evades xenophagy recognition primarily via its outer membrane protein B (OmpB), which blocks polyubiquitylation of bacterial surface proteins. Unlike pathogens that manipulate host actin to avoid ubiquitylation, OmpB acts locally to protect OmpA—a common target of host ubiquitin machinery—from autophagic recognition (35). Potential mechanisms include OmpB’s deubiquitylase activity, its abundance (over 10% of bacterial protein mass) enabling surface camouflage, recruitment of host proteins to cleave ubiquitin chains, or enzymatic modification of the bacterial surface required for recognition by the host ubiquitin machinery (40).

### 3.2 Interference with autophagy initiation

Autophagy, marked by autophagosome formation, is regulated by the ULK1 complex (ULK1, FIP200, ATG101), controlled by mTORC1 and AMPK (41). While AMPK activates ULK1, mTORC1 suppresses it via phosphorylation of Ser757 (41). *Salmonella enterica*, a facultative intracellular pathogen, employs its T3SS to inject effectors like SopB into host cells (42). In B cells, SopB’s phosphatase activity elevates PIP3 levels, activating the PI3K/AKT pathway (43). This triggers mTORC1, which inhibits ULK1 and blocks autophagosome formation. By suppressing autophagy initiation via this PI3K/AKT/mTORC1 axis, enabling *S. enterica* prolonged survival within B cells (44).

Tuberculosis, caused by *Mycobacterium tuberculosis* (Mtb), remains a global health threat, particularly in low- and middle-income countries (45). Mtb could evades xenophagy to survive in macrophages. Autophagy initiation relies on the ULK1 complex (ULK1, FIP200, ATG13, ATG101), which recruits the VPS34 complex (VPS34, BECLIN-1, VPS15, and ATG14L) to generate phosphatidylinositol 3-phosphate (PI3P), driving autophagosome formation. The produced PI3P then binds with PI3P-binding proteins such as WIPI2B and DFCP1, together with other proteins, leads to the formation and expansion of the autophagosome, eventually forming the complete autophagosome (46). AMPK activates ULK1 (via phosphorylation of Ser317/Ser777), while mTORC1 suppresses it by phosphorylating ULK1 Ser757, blocking the interaction between ULK1 and AMPK. This coordinated phosphorylation is important for ULK1 in autophagy induction (41). Mtb disrupts this balance using secreted acid phosphatase SapM (Rv3310). SapM enhances mTORC1 activity by dephosphorylating Raptor (a key mTORC1 component) at Ser792, countering AMPK’s inhibitory effect (47, 48). This sustains mTORC1 activation, preventing autophagy initiation and aiding bacterial survival (45).

### 3.3 Manipulation of autophagy machinery

*Staphylococcus aureus*, an opportunistic pathogen colonizing human skin and nares, subverts xenophagy to persist intracellularly (49). Its cell wall components can be detected as PAMPs and trigger autophagy via ubiquitination and receptor recruitment (e.g., p62, NDP52) in non-phagocytic cells (50, 51), while Galectin-8 targets damaged endosomes for autophagic clearance (52). In phagocytes, *S. aureus* initially resides in vesicles before lysosomal fusion (53), but certain strains block autophagy flux—disrupting LC3-II and p62—and alkalinise autolysosomes to create replication niches (54). Central to this evasion is the accessory gene regulatory (AGR) quorum-sensing system, which regulates toxins like α-toxin and phenol-soluble modulins (PSMα) (55). *S. aureus* secreted α-toxin to inhibit autophagosome-lysosome fusion, while PSMα aids phagosomal escape (56). AGR also dysregulates *S. aureus*-containing phagosomes maturation, preventing autophagosome acidification and LAMP-2 acquisition (57). In polymorphonuclear neutrophils, AGR-driven p53 accumulation driving transcriptional activation of pro-autophagic membrane protein damage-regulated autophagy monitor (DRAM), inducing autophagosome buildup to sustain survival niche for *S. aureus* (58). Additionally, *S. aureus* phosphorylates mitogen-activated protein kinase 14 (MAPK14) and ATG5, further blocking autophagosome maturation (50). By manipulating autophagy through AGR-dependent and -independent mechanisms, *S. aureus* evades lysosomal degradation, ensuring intracellular persistence.

*Salmonella enterica serovar Typhimurium* (*S. Typhimurium*), a Gram-negative intracellular pathogen, employs two virulence gene clusters—Salmonella Pathogenicity Island 1 (SPI1) and SPI2—to invade host cells and survive intracellularly (59). SPI1’s T3SS enables entry into nonphagocytic cells, while SPI2’s T3SS facilitates survival within SCVs by secreting effectors that block lysosomal fusion (60). These SPI2 effectors manipulate host signalling pathways, including sustained activation of AKT and mTOR, which suppress autophagy—a cellular recycling process controlled by mTOR, a nutrient-sensing kinase, activation of which forms two multiprotein complexes, mTORC1 and mTORC2 (61). Notably, the SPI2 effector SopB activate AKT at Ser473 via mTORC2 early in infection (62). Additionally, *S. Typhimurium* disrupts AMPK, a key energy sensor activated during ATP depletion. Although infection initially triggers AMPK activity due to low ATP levels, SPI2 targets the AMPK-activation complex (including Sirtuin-1 [SIRT1] and liver kinase B1) for lysosomal degradation, blunting AMPK’s role in autophagy. AMPK normally promotes autophagy by inhibiting mTOR or directly phosphorylating autophagy-related proteins like ULK1 (41). SIRT1 further regulates autophagy by deacetylating components of the autophagic machinery (e.g., ATG5, LC3) and interacting with AMPK in a feedback loop (63, 64). By hijacking these pathways—activating AKT/mTOR, degrading AMPK components, and disrupting SIRT1—SPI2-dependent mechanisms reduce autophagic flux in macrophages (65). This allows *S. Typhimurium* to evade host defenses, replicate within SCVs, and propagate systemic infection.

*Listeria monocytogenes* (*L. monocytogenes*), a Gram-positive intracellular pathogen, causes listeriosis—a severe foodborne illness. It enters the host via the gastrointestinal epithelium, either through M cells in Peyer’s patches or alternative routes. Once inside, dendritic cells and macrophages transport the bacteria to mesenteric lymph nodes and tissues, enabling systemic spread (66). To survive, *L. monocytogenes* rapidly escapes phagosomes using listeriolysin O (LLO), a pore-forming toxin, aided by phospholipases C enzymes PLCA and PLCB (67). LLO punctures the phagosomal membrane, creating pores that expand over time, allowing bacterial release into the cytosol (68, 69). These enzymes also disrupt autophagy by depleting PI3P, a key molecule for autophagosome formation (70, 71). Host factors like GILT (activating LLO via thiol reduction) and CFTR (altering phagosomal chloride levels) aid bacterial escape (72). Inefficient LLO activity leads to Spacious *Listeria*-containing Phagosomes (SLAPs)—enlarged, non-maturing compartments where bacteria persist and replicate slowly. By subverting xenophagy and hijacking host pathways, *L. monocytogenes* evades degradation, ensuring intracellular survival and systemic infection (73).

*Burkholderia pseudomallei* (*B. pseudomallei*), a Gram-negative intracellular pathogen, invades both phagocytic and non-phagocytic cells. Within 15 minutes of entry, it escapes endocytic vesicles via its TTSS3 and effector protein BOPA, avoiding lysosomal degradation (74, 75). BOPA contains two functional domains: a Rho GTPase inactivation domain and a cholesterol-binding domain (76, 77). The latter may disrupt lysosomal fusion by accumulating cholesterol on phagosomal membranes, akin to mechanisms seen in *Mycobacterium avium* infections (78). TTSS structural genes like BSAZ and effector BIPD are critical for timely vesicle escape, as mutants show delayed evasion of LAMP-1-positive vacuoles (79). Metabolic genes (PURM, PURN, HISF, PABB) and BPSL1528 further support intracellular replication (80). To survive, *B. pseudomallei* subverts xenophagy by upregulating miR-146a, which inhibits lysosomal acid lipase A, blocking autophagosome-lysosome fusion (81). It also suppresses ATG10 (essential for autophagy) via miRNAs (MIR4458, MIR4667-5p, MIR4668-5p), dampening autophagic activity. These strategies enable the pathogen to persist and thrive within host cells (82).

*Brucella*, a Gram-negative intracellular pathogen, comprises six species (*B. melitensis, B. abortus, B. suis, B. ovis, B. canis, B. neotoma*) with genetic similarity but varying host preferences and virulence (83). It survives within macrophages and non-phagocytic cells by hijacking xenophagy, forming *Brucella-*containing vacuoles (BCVs) that mature into endoplasmic reticulum-derived replicative niches (84). The T4SS secretion system delivers effectors (e.g., RICA, VCEA, BtpB) to manipulate host processes. VCEA and BTPB directly regulate autophagy, while host proteins like WIPI, ATG9, BECLIN1, and ATG14L aid BCV formation (85, 86). However, how *Brucella* precisely exploits autophagy machinery—or which bacterial factors control intracellular trafficking—remains unclear. By subverting lysosomal fusion and leveraging autophagosome-like structures, the pathogen ensures survival and proliferation within host cells.

### 3.4 Hijacking autophagy for replication

*Francisella* species thrive in the cytosol of diverse host cells and organisms. After internalization, the bacteria transiently occupy a vacuole marked by early and late endosome markers before escaping into the cytosol—a critical step for rapid replication, mediated by genes in the *Francisella* pathogenicity island (homologous to TVISS) (87). Autophagy, triggered by infection via an ATG5-independent pathway, supplies amino acids and carbon for bacterial metabolism. Inhibiting autophagy stalls *Francisella* growth, but adding non-essential amino acids or pyruvate restores replication, confirming their role as nutrients (88). During infection, bacteria localize near autophagosomes in host cells, suggesting exploitation of autophagy for resources. Interestingly, late-stage infection in murine macrophages reveals large autophagic *Francisella*-containing vacuoles (FCVs), dependent on bacterial protein synthesis (89). However, FCVs form only in mouse cells, not human macrophages, leaving their biological relevance uncertain (90). Identifying bacterial factors that manipulate autophagy could clarify how *Francisella* hijacks this process for nutrition.

*Brucella* spp. persist within membrane-bound BCVs, hijacking the host secretory pathway to transform their initial endosomal BCV into an ER-derived replicative BCV. Critical to this process is the VIRB T4SS, which injects bacterial effectors to manipulate host functions (91, 92). Following internalization, BCVs acquire early and late endosomal markers (e.g., LAMP-1) and transiently interact with lysosomes via RAB7 (93). However, the BCV eventually diverts to ER exit sites (ERES), excluding endosomal markers. This shift relies on Sar1 and Rab2 GTPases, which regulate ERES integrity and Golgi-ER transport (94, 95). *Brucella* infection also activates the unfolded protein response (UPR), a stress pathway restoring ER homeostasis (96). While *B. melitensis* triggers all three UPR arms (IRE1α, protein kinase RNA-like ER kinase, and activating transcription factor 6-dependent pathways) via effector TCPB. *B. abortus* and *B. suis* primarily activate IRE1α, linked to host detection of T4SS activity (97, 98). IRE1α activation—dependent on host protein YIP1A—upregulates Sar1 and COPII components, enhancing ERES function and ER membrane acquisition for BCV biogenesis (99). This process intersects with autophagy: IRE1α-driven UPR stimulates ATG9- and WIPI-dependent autophagosome formation, which Brucella exploits to establish its replicative BCV (99). Despite progress, the exact interplay between UPR, autophagy, and BCV formation remains unclear, particularly how ER stress benefits *Brucella*’s intracellular survival.

The *Chlamydiaceae* family comprises Gram-negative, obligate intracellular bacteria that infect humans and animals (100). Among these, *Chlamydia trachomatis* (*C. trachomatis*) primarily targets the human female genital tract. During infection, the pathogen establishes a protective vacuole called the inclusion, where it replicates (101). Evidence suggests *C. trachomatis* hijacks autophagosomes—either as nutrient sources or transport vehicles—to support its survival (102). A key player here is LC3, a protein central to autophagy. Normally, LC3-I (cytosolic) converts to lipid-bound LC3-II, marking autophagosomal membranes. However, *C. trachomatis* repurposes LC3 in an autophagy-independent manner: the protein stabilizes the host cell’s microtubule network, which is critical for bacterial inclusion stability and movement. Depleting LC3—even in autophagy-deficient cells—severely hampers *C. trachomatis* growth, underscoring its exploitation of LC3 beyond conventional autophagy pathways. Essentially, the pathogen co-opts LC3’s structural role to anchor inclusions to the cytoskeleton, ensuring its replication niche remains intact (103).

*Coxiella burnetii*, the Gram-negative bacterium behind Q fever, thrives by hijacking host xenophagy to form its replication niche, the *Coxiella*-containing vacuole (CCV) (104). Stimulating autophagy—via nutrient deprivation or rapamycin—boosts bacterial replication and enlarges the CCV, while blocking autophagy disrupts CCV development (105). Key autophagy genes (TFEB/TFE3, ATG proteins, STX17) enable homotypic vacuole fusion, forming expansive CCVs (106). The pathogen delays lysosomal protease delivery (e.g., cathepsin D) and exploits autophagy machinery, increasing lipidated LC3B-II and stabilizing p62—a cargo receptor degraded during normal autophagy (107, 108). This manipulation relies on bacterial effectors secreted via the T4SS (109). Critical effectors like CVPB (CIG2) label CCVs, recruit autophagosomal LC3, and sustain phosphatidylinositol 3-phosphate to maintain autolysosomal maturation (110, 111). CVPB, alongside CVPC-E, ensures CCV-LAMP1 vesicle fusion, vital for replication (112). Similarly, CVPF interacts with RAB26—a GTPase regulating autophagy—by binding its inactive form, possibly acting as a GEF/GDF to recruit RAB26 to CCVs. Active RAB26 collaborates with ATG16L1 to stimulate LC3 lipidation, enhancing autophagosome-CCV fusion (113). Depleting RAB26 reduces LC3 recruitment and CCV size, impairing bacterial growth. Another effector, CIG57, recruits clathrin to CCVs, indirectly supporting LC3B association (108). Together, these effectors orchestrate RAB GTPase activity and autophagy flux, diverting host membranes and nutrients to expand CCVs (112). In short, *C. burnetii*’s T4SS effectors co-opt autophagy regulators (e.g., RAB26, clathrin) to stabilize its niche, illustrating how intracellular pathogens rewire vesicular trafficking for survival.

Bacteria of the genus *Yersinia* cause illnesses ranging from enteritis to plague. *Y. enterocolitica*, a species with diverse strains classified by biochemical profiles and O-antigen serotyping, manipulates autophagy to create a protective niche. After invading host cells, it occupies vacuoles resembling autophagosomes but actively blocks lysosomal fusion and acidification, enabling survival and replication (114, 115). Studies show *Yersinia* persists in intestinal macrophages during early infection and replicates within autophagosomes in macrophages by halting maturation (116, 117). Disrupting autophagy forces bacteria into acidic compartments for degradation, confirming autophagy’s role in sustaining their niche (116). Similarly, *Y. pseudotuberculosis* exploits arrested autophagosomes, likely fueling replication via nutrient-rich autophagosomal membranes. *Staphylococcus aureus* also hijacks xenophagy. In HeLa cells, it replicates within LC3-positive autophagosomes for 3–12 hours before escaping into the cytosol to trigger apoptosis. Autophagy-deficient cells (e.g., ATG5 knockouts) prevent bacterial replication, as phagosome-lysosome fusion resumes. Notably, AGR mutants—which lack virulence gene expression—fail to induce autophagy and cannot survive intracellularly (57).

## 4 Xenophagy in aquaculture

Xenophagy, a selective form of autophagy targeting intracellular pathogens, is particularly critical in combating bacterial infections prevalent in aquatic animals, such as those caused by *Aeromonas hydrophila*, *Edwardsiella*, *Mycobacterium*, and so on (as shown in **Table 2**).

**Table 2:** Bacteria regulated autophagy genes/proteins in teleost species.

Species	Bacteria	Autophagy-related genes/Proteins
Zebrafish (118, 119)	*Mycobacterium*, *Shigella flexneri*, *Mycobacterium marinum*, *Staphylococcus aureus*, *Salmonella Typhimurium*	atg5, atg7, lc3, p62/sqstm1, dram1,
Common Carp (120)	*Aeromonas hydrophila*	atg12, atg5,mtor,lc3, beclin1
Grass Carp (*Ctenopharyngodon idella*) (121, 122)	*Escherichia coli*, *Edwardsiella piscicida*	Atg5, Atg16l1,lc3
Yellow catfish (*Pelteobagrus fulvidraco*) (123)	*Aeromonas hydrophila, Edwardsiella tarda*	Atg5,Atg7
Japanese flounder (Paralichthys olivaceus) (124)	*Edwardsiella tarda*	Atg7
fathead minnow (*Pimephales promelas*) (125)	*Vibrio parahaemolyticus*	Lc3

In teleosts, xenophagy is integral to innate immunity. For instance, zebrafish macrophages utilize xenophagic pathways to degrade intracellular pathogens like *Shigella flexneri* and *Salmonella Typhimurium*, with autophagy-related genes (ATG5, p62, DRAM1) being essential for bacterial clearance (126). However, certain pathogens subvert these mechanisms (127). *Edwardsiella piscicida*, a Gram-negative bacterium, induces mitophagy in fish monocytes/macrophages, promoting its intracellular survival by degrading damaged mitochondria and evading antimicrobial ROS (128). This pathogen downregulates host autophagy regulators (e.g., ATG16L1) and PRRs such as NOD1, impairing immune detection and facilitating replication (129, 130). Similarly, *Legionella pneumophila* manipulates autophagosome maturation, exploiting endoplasmic reticulum (ER)-derived vacuoles to avoid lysosomal degradation, while *Mycobacterium marinum* recruits autophagy proteins (e.g., LC3) via its ESX-1 secretion system to create replication-permissive compartments (131–133). Pathogen effector proteins further illustrate this subversion. *Vibrio parahaemolyticus* secretes VopQ, which blocks autophagic flux, impairing inflammasome activation and enabling immune evasion (134). Conversely, *Vibrio harveyi* exploits host eukaryotic translation initiation factor 3k to degrade MyD88, a key mediator of NF-κB signalling, thereby suppressing inflammatory responses (135, 136). Such strategies highlight the complex interplay between xenophagy and bacterial survival, where pathogens either inhibit autophagic degradation or co-opt its machinery to establish infection niches. Notably, the role of xenophagy varies contextually. While it restricts pathogens like *Edwardsiella tarda* via the pol-miR-3p-2-p53-BECLIN1 axis in Japanese flounder (137), it paradoxically aids *Staphylococcus aureus* replication in zebrafish neutrophils by forming non-acidified LC3-positive phagosomes (138). These findings underscore the dual nature of xenophagy in aquatic immunity and pathogenicity. Advancing disease management in aquaculture requires elucidating molecular mechanisms underlying xenophagy-pathogen interactions, including effector protein functions, PRR modulation, and organelle-specific autophagy (e.g., mitophagy). Targeted therapeutic strategies, such as enhancing autophagic flux or blocking pathogen-mediated subversion, hold promise for mitigating infections. Advancing autophagy research in aquatic species requires specialized tools. While DNA, RNA, and protein-based assays adapted from mammalian systems exist, most are only validated in zebrafish. Key RNA techniques include CRISPR/Cas9, TALENs, and morpholino knockdowns, while transcript analysis uses qRT-PCR and sequencing, and protein studies employ WB and TEM (139–141). However, species-specific reagents and protocols for non-model teleosts are lacking, and dynamic autophagy studies face financial and technical hurdles. Developing standardized assays for diverse teleosts remains a critical need. Future research should prioritize *in vivo* models and multi-omics approaches to unravel these complexities, ultimately informing sustainable aquaculture practices.

## 5 Conclusion and future perspectives

Xenophagy, a conserved eukaryotic mechanism, functions dually in host defense and pathogen exploitation. While it typically degrades intracellular microbes, certain bacteria subvert xenophagy via effector proteins to inhibit lysosomal fusion, hijacking autophagosomes as replicative niches. This interplay is pathogen- and cell type-dependent, highlighting xenophagy’s complex role in infection dynamics—critical for developing targeted therapies amid rising antibiotic resistance. In teleost, autophagy regulates physiological and pathological processes through pathways analogous to mammals, offering potential as a biomolecular marker or therapeutic target in teleosts. Although research on autophagy in teleost remains limited, progress has been made in identifying autophagy-inducing conditions, genes, and pathogen-triggered autophagic responses. Transgenic zebrafish and cell lines have been instrumental in studying autophagy regulation and its role in anti-pathogen defense. With advancing genetic and imaging tools, zebrafish will continue to enhance our understanding of autophagy in bacterial immunity. Additionally, due to their aquatic environment, teleost lysosome-autophagy systems serve as sensitive biomarkers for ecosystem health monitoring (142). Beyond theoretical significance, autophagy research in teleost holds practical value, offering insights into disease mechanisms and potential applications for aquaculture benefits.

## Glossary

ActA: Actin assembly-inducing protein
Agr: accessory gene regulatory
AMPK: adenosine monophosphate-activated protein kinase
AKT: serine/threonine protein kinase B
Arp2/3 complex: Actin-related protein 2/3 complex
ATG5: autophagy related 5
Atg16L1: Autophagy related 16 like 1
BCVs: *Brucella-*containing vacuoles
BORC: BLOC-one-related complex
*B. pseudomallei*: *Burkholderia pseudomallei*
CCV: *Coxiella*-containing vacuole
CFTR: cystic fibrosis transmembrane conductance regulator
CRISPR/Cas9: Clustered regularly interspaced palindromic repeats associated protein 9;*C. trachomatis*, *Chlamydia trachomatis*
CvpB: Coxiella vacuolar protein B
DAMPs: damage-associated molecular patterns
DFCP1: Double FYVE containing protein 1
DRAM: damage-regulated autophagy monitor
ERES: ER exit sites
ERK: extracellular signal-regulated kinase
ESAT-6: early secretory antigenic target 6
ESX-1: ESAT-6 system 1
ESPB: ESX-1 secretion-associated protein B
FCVs: *Francisella*-containing vacuoles
FIP200: focal adhesion kinase family interacting protein of 200 kD
GAS: *Group A Streptococcus*
GILT: Gamma-interferon-inducible lysosomal thiol reductase
IcsB: N-epsilon-fatty acyltransferase IcsB
IFN-γR1: IFN-γ receptor 1
InlK: Internalin K
IRE1α: Inositol-requiring transmembrane kinase/endonuclease-1
LAP: LC3-associated phagocytosis
LAMP-2: Lysosome-associated membrane protein-2
LC3: microtubule-associated protein 1 light chain 3
*L. monocytogenes*: *Listeria monocytogenes*
LLO: listeriolysin O
*L. monocytogenes*: *Listeria monocytogenes*
MAPK14: mitogen-activated protein kinase 14
Mitophagy: mitochondria autophagy
Mtb: *Mycobacterium tuberculosis*
mTOR: mechanistic target of rapamycin kinase
mTORC1: mechanistic target of rapamycin complex 1
NBR1: neighbor of BRCA1 gene 1
NDP52: nuclear dot protein, 52 kDa
OmpB: outer membrane protein B
PAMPs: pathogen-associated molecular patterns
Pexophagy: peroxisomes autophagy
PI3P: phosphatidylinositol 3-phosphate
PIP3: Phosphatidylinositol (3,4,5)-trisphosphate
PSMα: phenol-soluble modulins α
PRRs: pattern recognition receptors
PRKN: parkin RBR E3 ubiquitin protein ligase
PTPA: Protein phosphatase 2A phosphatase activator
*R. parkeri*: *Rickettsia parkeri*
Ribophagy: ribophagy autophagy
SapM: Secretion of an Acid Phosphatase of *M. tuberculosis*
SCV: *Salmonella* -containing vacuole
*S. flexneri*: *Shigella flexneri*
SLAPs: Spacious *Listeria*-containing Phagosomes
SMURF1: SMAD specific E3 ubiquitin protein ligase 1
SpeB: Streptococcal pyrogenic exotoxin B
*S. Typhimurium*: *Salmonella enterica serovar Typhimurium*
SPI1: Salmonella Pathogenicity Island 1
SIRT1: Sirtuin-1
SQSTM1: sequestosome 1
STAT1: signal transducer and activator of transcription 1
STX17: syntaxin 17
TALENs: transcription activator-like effector nucleases
TBK1: TANK-binding kinase 1
TEM: Transmission electron microscopy
TFEB: transcription factor EB
T3SS: type III secretion system
TLR2: Toll-like receptors 2
ULK1: Unc-51-like kinase 1
UPR: unfolded protein response
VASP: vasodilator-stimulated phosphoprotein
VPS34: vacuolar protein sorting 34
WB: western blot
WIPI: WD-repeat protein interacting with phosphoinositides
Xenophagy: pathogens autophagy.
